# Postmastectomy Functional Impairments

**DOI:** 10.1007/s11912-023-01474-6

**Published:** 2023-11-13

**Authors:** Eden Marco, Gabrielle Trépanier, Eugene Chang, Emma Mauti, Jennifer M. Jones, Toni Zhong

**Affiliations:** 1 Temerty Faculty of Medicine, University of Toronto, Toronto, ON Canada; 2 Department of Medicine, Division of Physical Medicine & Rehabilitation, University of Toronto, Toronto, ON Canada; 3 Department of Supportive Care, Cancer Rehab & Survivorship Program, Princess Margaret Cancer Centre, Toronto, ON Canada; 4 Multisystem & Musculoskeletal Rehabilitation Program, Toronto Rehabilitation Institute, Toronto, ON Canada; 5 Cancer Rehabilitation and Survivorship Program, Department of Supportive Care, Princess Margaret Cancer Centre, Toronto, ON Canada; 6 Department of Psychiatry, University of Toronto, Toronto, ON Canada; 7 Division of Plastic and Reconstructive Surgery, Department of Surgery, University of Toronto, Toronto, ON Canada

**Keywords:** Functional impairment, Postmastectomy, Breast cancer survivorship

## Abstract

**Purpose of Review:**

This narrative review aims to offer a thorough summary of functional impairments commonly encountered by breast cancer survivors following mastectomy. Its objective is to discuss the factors influencing these impairments and explore diverse strategies for managing them.

**Recent Findings:**

Postmastectomy functional impairments can be grouped into three categories: neuromuscular, musculoskeletal, and lymphovascular. Neuromuscular issues include postmastectomy pain syndrome (PMPS) and phantom breast syndrome (PBS). Musculoskeletal problems encompass myofascial pain syndrome and adhesive capsulitis. Lymphovascular dysfunctions include lymphedema and axillary web syndrome (AWS). Factors such as age, surgical techniques, and adjuvant therapies influence the development of these functional impairments.

**Summary:**

Managing functional impairments requires a comprehensive approach involving physical therapy, pharmacologic therapy, exercise, and surgical treatment when indicated. It is important to identify the risk factors associated with these conditions to tailor interventions accordingly. The impact of breast reconstruction on these impairments remains uncertain, with mixed results reported in the literature.

## Introduction

Breast cancer is the most frequently diagnosed cancer on a global scale [1, 2]. It is projected that the United States will experience approximately 300,590 newly reported cases in 2023, with an overwhelming majority affecting females [1]. Surgical intervention is the widely adopted and established standard of care for managing most early-stage breast cancers, offering diverse options such as breast-conserving surgery, unilateral or bilateral mastectomy, and choices regarding immediate, delayed, or no reconstruction [3]. In cases where the tumor is large relative to the breast size, there are multiple tumors in different areas of the breast, or if the individual has previously undergone radiation therapy to the breast, mastectomy is the accepted practice [3]. Recent statistics from 2020 emphasize a substantial proportion of women diagnosed with early-stage breast cancer in the United States who underwent bilateral mastectomy. Among women aged 31 to 40 years, 33.0% underwent this procedure, while for those aged 30 or younger, the percentage was even higher at 39.9% [4]. Age plays a significant role in the choice of mastectomy as a treatment option, with younger women more likely to opt for this procedure compared to older individuals [4].

**Fig. 1:**
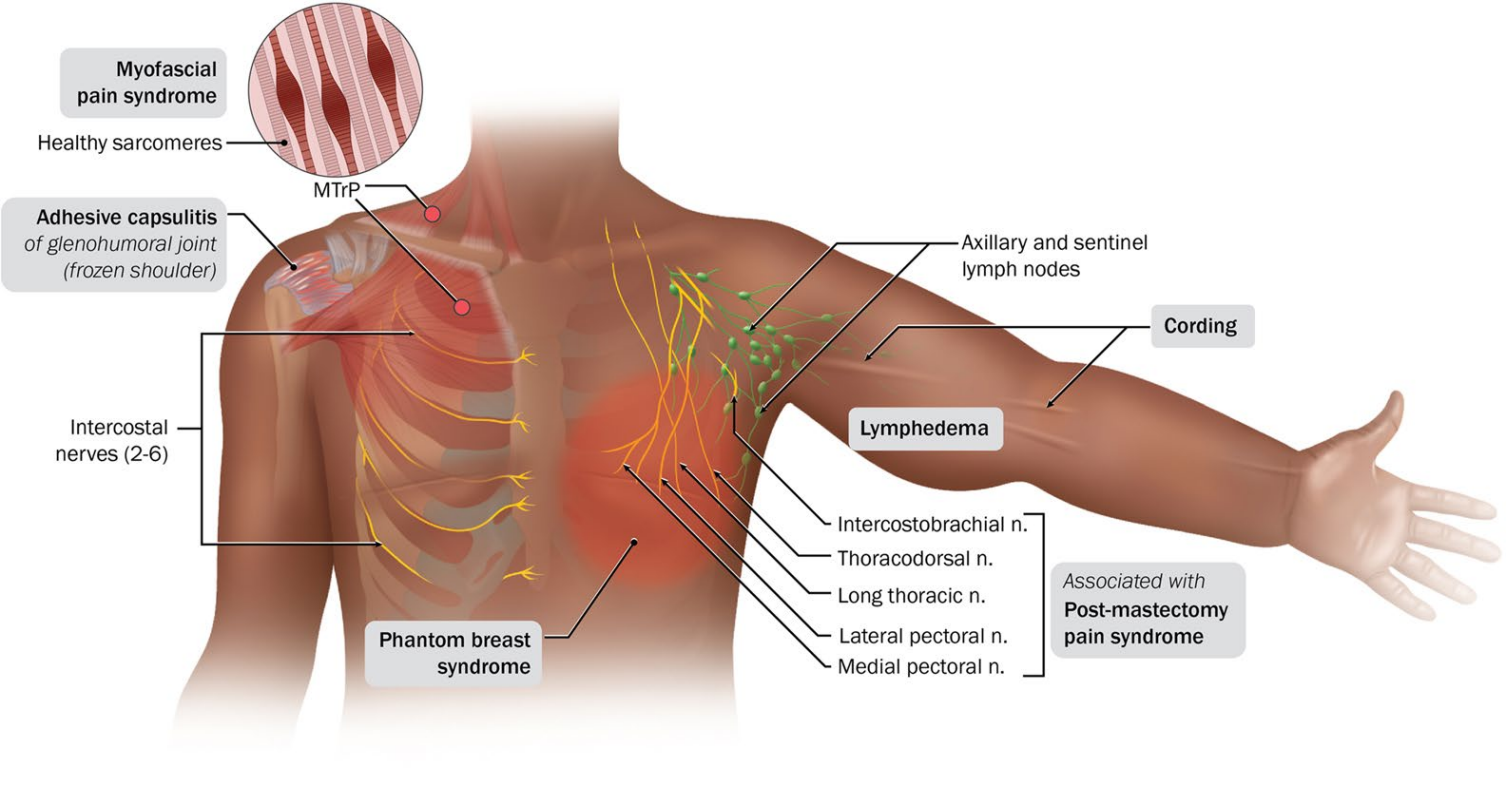
Anatomical representation of functional impairments

Although bilateral mastectomy is an effective treatment option, many patients encounter both short-term and long-term postmastectomy sequelae [3]. These include various neuromuscular, musculoskeletal, and lymphovascular issues, each with distinct characteristics and implications for patient well-being [5]. Neuromuscular issues involve the nerves and/or muscles in the affected area. Nerve damage during surgery or the removal of lymph nodes can result in symptoms such as pain, numbness, tingling, or weakness [3, 5, 6]. Common neuromuscular conditions include postmastectomy pain syndrome (PMPS), which involves chronic pain persisting beyond the expected healing period, and phantom breast syndrome (PBS), which describes the perception of pain or sensations in the breast area following mastectomy [3, 5, 7••]. Musculoskeletal issues encompass the bones, joints, and surrounding connective tissue. These conditions can manifest in various ways, including myofascial pain syndrome, which involves the development of trigger points or muscle “knots” that cause both localized and referred pain [3, 5, 7••]. Additionally, adhesive capsulitis, widely recognized as frozen shoulder, is a well-documented condition that causes stiffness and restricted mobility in the shoulder joint [3, 5, 7••]. Lymphovascular impairments are associated with the lymphatic system and blood vessels. Lymphedema is a common consequence of lymph node removal during mastectomy and is characterized by swelling due to fluid buildup [3, 5, 7••, 8]. Another common lymphovascular condition is axillary web syndrome (AWS), also known as cording, where visible or palpable fibrous cords develop in the axilla, causing pain and limited movement [3, 5, 8]. Figure 1 provides an anatomical representation of functional impairments discussed in this review.

The prevalence of postmastectomy functional impairments can be increased by adjuvant therapies, such as radiation, chemotherapy, and endocrine treatments [3, 9]. Moreover, the inclusion of sentinel lymph node biopsy (SLNB) and axillary lymph node dissection (ALND) during mastectomy, primarily performed for staging, can influence the likelihood of developing certain long-term sequelae [3, 9]. Despite the potential for sequelae, mastectomy remains a favorable option overall. Recent studies suggest that, in comparison to lumpectomy, mastectomy has fewer post-operative side effects and is associated with less chronic pain [10].

With advancements in medical therapies leading to lower mortality rates and greater 5-year survival rates for breast cancer patients (90.8%, 95% CI—90.5% to 91.1%), enhancing the long-term quality of life (QoL) for this population is of utmost importance [2]. It is essential to identify and understand the functional impairments that may arise in breast cancer patients following mastectomy, as these conditions often hinder one’s ability to carry out activities of daily living. Equally important is gaining knowledge about the diverse management strategies and interventions available to effectively address these impairments [5]. Notably, there can be an overlap between these categories, and some patients may experience concurrent issues [5]. By distinguishing between these categories, healthcare professionals can develop optimized treatment plans and support patients throughout their recovery journey, ultimately leading to an improved QoL [5]. This article provides evidence-based resources to enhance understanding of the various functional impairments endured by mastectomy patients.

## Neuromuscular

### Postmastectomy Pain Syndrome (PMPS)

PMPS was first documented in the 1970s and continues to be an enduring functional impairment, affecting 20–68% of mastectomy patients [2, 11, 12]. It is characterized by persistent dull, burning, and aching sensations that affect the chest, axilla, and the arm on the side where the mastectomy was performed for a period of at least 3–6 months following surgery. However, the duration of this period may vary slightly [2, 11, 13]. The most common cause of PMPS is thought to be due to intercostobrachial nerve damage and subsequent neuroma formation from surgical dissection, with a higher likelihood in cases where mastectomies are performed alongside ALND [5, 7••, 13]. However, PMPS is also associated with injury to other regional peripheral nerves including the medial pectoral, lateral pectoral, thoracodorsal, long thoracic nerves, and intercostal nerves II-VI [11, 13] (Fig. 1). Risk factors include younger age, higher body mass index (BMI), concurrent radiotherapy treatment, lack of support from family and friends, and belonging to racial/ethnic minorities [2, 3, 12]. In Wang et al.’s meta-analysis of 30 studies and 19,813 postmastectomy patients, it was found that the odds of PMPS increased with decreasing age. Specifically, for every 10-year decrease in age, the odds ratio was 1.36 (95% CI = 1.24—1.48) [14]. Though the exact reason remains unclear, it has been proposed that this inverse relationship may be due increased pain receptor sensitivity and risk of nerve damage in younger individuals, as well as a greater likelihood of having a tumor with high histopathological grading, delayed diagnosis, and receiving a more aggressive treatment regimen [2].

Patients with persistent PMPS report significantly lower QoL compared to those for which PMPS has resolved, which points to a need for survivorship and rehabilitation measures [2]. There is considerable evidence suggesting the effectiveness of physical therapy in treating pain associated with PMPS. Guidelines generally recommend initiating exercises as early as one day after surgery, with an initial emphasis on gentle range of motion (ROM) movements. Over a period of 6–8 weeks, the regimen progresses to include strengthening exercises, ultimately aiming to restore full ROM [2]. In their recent meta-analysis, Kannan et al. demonstrated statistically significant benefits of incorporating exercise to improve both pain and overall QoL for mastectomy-treated breast cancer patients. However, the type of exercise interventions and their specific parameters varied greatly among the reviewed trials, which included resistance training, land-based and water-based aerobic exercise, low-intensity walking, and stretching [11]. Other treatment strategies are also available for PMPS. Chappell et al. revealed 10 major treatment modalities for PMPS in their systematic review. These included fat grafting, neuroma surgery, lymphedema surgery, nerve blocks and neurolysis, laser, antidepressants, neuromodulators, physical therapy, mindfulness-based cognitive therapy, and capsaicin [15•]. Autologous fat grafting stands out as a highly effective treatment modality for PMPS, supported by strong evidence from the reviewed studies [15•]. This is largely due to the regenerative properties of adipose tissue which contains adipose-derived stem cells involved in secreting pro-survival cytokines and growth factors [16]. In addition, both amitriptyline and venlafaxine, two commonly used antidepressants, have demonstrated significant effectiveness in reducing PMPS-associated pain [17]. For those who choose not to take medication or do not benefit from it, peripheral blocks offer an effective alternative [18] (Table 1). Given the wide range of treatment options available, the choice of therapy for PMPS should ultimately be based on personal preference and individual circumstances, with an emphasis on adopting a multimodal and multidisciplinary approach to effectively manage symptoms [15•].

**Table 1:** Summary of functional impairments and management strategies

Functional Impairments	Management
Neuromuscular	
Postmastectomy pain syndrome	Physical therapy, exercise, fat grafting, neuroma surgery, lymphedema surgery, nerve blocks and neurolysis, laser, antidepressants, neuromodulators, physical therapy, mindfulness-based cognitive therapy, capsaicin
Phantom breast syndrome	Eye Movement Desensitization and Reprocessing (EMDR), nerve blocks, nerve stabilizers, analgesic agents
Musculoskeletal	
Myofascial pain syndrome	Mobility exercises, stretching, strengthening, myofascial release, physical therapy, local anesthetic injections, dry needling, electrical stimulation, acupuncture, NSAIDs, muscle relaxants, benzodiazepines, sertonin and norepinephrine reuptake inhibitors (SNRIs), tricyclic antidepressants, menthol, cannabidiol, lidocaine-containing creams
Adhesive capsulitis	NSAIDs, acetaminophen, intra-articular corticosteroid injections, passive mobilization exercises, stretching, electrotherapy, hyaluronic acid injections, platelet-rich plasma injections, hydrodistention, extracorporeal shockwave therapy, low-level laser therapy, calcitonin, surgical intervention
Lymphovascular	
Lymphedema	Complete Decongestive Therapy (CDT) involving manual lymphatic drainage (MLD), multiple layer compression bandaging, exercise, and proper skin care. Pneumatic compression devices, surgical procedures including debulking, lymphovenous anastomosis, vascular lymph node transplantation
Axillary web syndrome	Physical therapy, exercise, myofascial release, soft tissue mobilization, cord manipulation, stretching while arm is abducted, analgesics, NSAIDs, proangiogenic drugs, surgical intervention

### Phantom Breast Syndrome (PBS)

PBS is characterized by the occurrence of pain or nonpainful sensations such as itching or tingling in the amputated breasts [3, 19•] (Fig. 1). A crucial factor in distinguishing phantom breast pain from other forms of pain is the exclusive presence of pain in the absent breast, without any pain reported in the ipsilateral chest wall or arm [7••]. This phenomenon can manifest intermittently or persistently, appearing months to years after mastectomy, and has been reported in varying percentages of postmastectomy patients, ranging from 1 to 66% [19•]. PBS is believed to stem from changes in the central nervous system and nerve damage resulting from surgery and has been shown to be associated with the weight of breast tissue removed [7••, 20]. Based on a recent study by Viscone and Weyandt, the prevalence rates of PBS have shown a decline, ranging from 0 to 19%. Moreover, the majority of individuals described their experiences of pain or sensation as relatively mild [19•]. This decline in prevalence can be attributed to advancements in surgical techniques aimed at minimizing the risk of nerve damage during mastectomy [19•].

Recent studies have given limited attention to PBS treatments due to declining prevalence rates [19•]. However, there are a few potential interventions have been proposed to address this postmastectomy impairment. Eye Movement Desensitization and Reprocessing (EMDR) has shown promise in treating phantom pain in amputees and may be effective for PBS [21]. Grounded in Shapiro's Adaptive Information Processing model, which explains how traumatic experiences hinder natural information processing and contribute to psychological disorders, EMDR utilizes techniques such as eye movements or tapping to facilitate the processing of distressing memories, reduce their emotional impact, and promote healing [21]. Continuous paravertebral nerve blocks with ropivacaine have also demonstrated effectiveness in managing PBS symptoms in a randomized, placebo-controlled clinical trial [22]. Other treatment options include nerve stabilizers and analgesic agents [5] (Table 1). Further research is needed to explore preventive therapies and pain treatments for PBS, as this condition continues to affect the QoL of breast cancer survivors.

Nerve preservation in mastectomy is an emerging surgical technique aimed at preventing pain and restoring normal sensation in the breast. This procedure focuses on preserving or reconstructing nerves using allograft technologies to avoid nerve damage [23••]. Recent studies have shown promising results, with one study reporting preserved nipple/areolar complex sensation in 87% of breasts and no cases of dysesthesias or neuromas, indicating the potential of nerve-sparing mastectomies in preventing long-term pain and abnormal sensation [24].

## Musculoskeletal

### Myofascial Pain Syndrome

Myofascial pain syndrome is a condition characterized by the presence of myofascial trigger points (MTrPs), leading to localized pain [7••]. It has been found to affect as many as 45% of breast cancer patients [25]. MTrPs are found within taut muscular bands in the myofascial tissues and usually elicit pain when compressed, stretched, or overloaded [7••, 25] (Fig. 1). These trigger points can develop after surgery, causing localized pain and tenderness, reduced ROM, and referred pain in specific referral patterns [7••]. Factors such as muscle fibrosis resulting from inflammation, fascial dysfunction, and heightened excitability of motor nerves contribute to myofascial pain syndrome, all of which can occur after surgery [15•, 25]. Interestingly, active MTrPs have also been found in several muscles of patients with PMPS [15•]. In postmastectomy patients, active MTrPs are commonly located in the muscles of the shoulder girdle, specifically in the latissimus dorsi, serratus anterior, pectoralis major, infraspinatus, and upper trapezius muscles [7••, 25].

In a cross-sectional study of 64 breast cancer patients, it was found that, when compared to an untreated control group of breast cancer patients, those that received a mastectomy or lumpectomy had a significantly greater proportion of active MTrPs. No statistical difference was noted between the two surgery groups; however, the location of the MTrPs differed. In the lumpectomy group, the pectoralis major and infraspinatus muscles had the most active MTrPs. In the mastectomy group, the pectoralis major and upper trapezius muscles showed the majority of active MTrPs [25].

Various treatment modalities have been identified for patients with myofascial pain syndrome. The main objective is to gradually alleviate tension in the affected regions, with emphasis on massage techniques and customized physical therapy interventions [25]. Multiple interventions have shown efficacy in managing pain and alleviating trigger points among breast cancer patients. A comprehensive physical therapy program incorporating exercises, targeted massage sessions, and techniques such as mobility exercises, stretching, strengthening, and myofascial release, yielded notable reductions in neck and shoulder/axillary pain over an eight-week period [25]. Moreover, ultrasound-guided injections for trigger points in the internal rotator muscles of the shoulder have been shown to decrease pain intensity and improve shoulder ROM [25]. Additional approaches, including local anesthetic injections, dry needling, electrical stimulation, and acupuncture, have also demonstrated effectiveness in providing relief from chronic pain associated with trigger points [7••, 25].

Pharmacological management of myofascial pain syndrome includes the use of non-steroidal anti-inflammatory drugs (NSAIDs), muscle relaxants, benzodiazepines, serotonin and norepinephrine reuptake inhibitors (SNRIs), and tricyclic antidepressants [7••]. Topical treatments such as menthol, cannabidiol, or lidocaine-containing creams have also been used to improve symptoms [7••] (Table 1).

### Adhesive Capsulitis (Frozen Shoulder)

Adhesive capsulitis, commonly known as frozen shoulder, is characterized by pain and significant loss of both passive and active ROM in the glenohumeral joint [5, 7••, 26] (Fig. 1). Postmastectomy patients frequently experience shoulder morbidity, affecting anywhere from 1 to 68% of individuals [26]. Restricted ROM can arise due to inflammation and subsequent fibrosis leading to tightening of the glenohumeral joint [7••]. Adhesive capsulitis is often regarded as a self-limiting disorder that follows a typical progression through three distinct phases [27, 28]. The first stage, also referred to as the painful freezing stage, lasts for 2 to 9 months. During this phase, individuals experience sharp, diffuse shoulder pain that tends to worsen at night, as well as a gradual increase in stiffness. The pain begins to lessen as adhesive capsulitis enters the second stage, the frozen stage, which normally lasts between 4 and 12 months, while stiffness and loss of ROM in the glenohumeral joint are at their highest. The third stage, sometimes known as the "thawing stage," involves a gradual regaining of ROM and can take anywhere between 5 months and 2 years to complete [27, 28]. While adhesive capsulitis may resolve on its own, investigations suggest that a sizable fraction (20% to 50%) of patients have symptoms that last longer than two years [29]. Factors such as age (50 to 59 years), breast reconstruction, lymphedema, lymph node dissection, and aromatase inhibitor therapy may independently contribute to the risk of developing adhesive capsulitis [7••, 29]. Although mastectomy itself does not directly cause damage to the glenohumeral joint, the accompanying pain, tightness in the pectoral muscles, and changes in biomechanics can result in protective postures that place stress and tension on the joint capsule. This can lead to restricted mobility and the subsequent development of secondary adhesive capsulitis [29, 30].

The treatment approach for adhesive capsulitis involves addressing both ROM improvement and pain management. While NSAIDs or acetaminophen can be used to treat initial pain, intra-articular corticosteroid injections administered directly into the glenohumeral joint have demonstrated great effectiveness in alleviating adhesive capsulitis-related pain and improving ROM in both the short and long term [7••, 31]. Further enhancements in treatment outcomes have been observed when these injections are combined with a home exercise program in the later stages of adhesive capsulitis that includes passive mobilization, stretching, and electrotherapy [7••, 31]. The inclusion of progressive banded strengthening exercises and scapular stabilization maneuvers have also been shown to improve shoulder ROM and overall QoL in postmastectomy patients [28]. Other therapies include hyaluronic acid injections, platelet-rich plasma injections, hydrodistention, extracorporeal shockwave therapy, low-level laser therapy, and calcitonin [32, 33]. Surgical procedures, such as manipulation under general anaesthetic or arthroscopic capsular release, may be explored after other therapies have failed [32] (Table 1).

## Lymphovascular

### Lymphedema

Lymphedema is characterized by limb swelling, heaviness, tightness, restricted mobility, and, in certain instances, pain resulting from impaired lymphatic system function [3, 7••] (Fig. 1). Breast cancer-related postmastectomy lymphedema is a well-recognized phenomenon, with an incidence ranging from 8 to 52% within the initial two years following surgery. Notably, approximately 75% of cases manifest within the first year [34]. The variation in incidence rates can be partially attributed to the lack of standardized criteria for defining and measuring lymphedema [35]. A recent retrospective analysis identified several key risk factors for lymphedema-related events occurring within two years after mastectomy. These factors include higher comorbidity levels at baseline, longer hospitalization duration, more recent mastectomy procedures, higher BMI, younger age, non-Asian race, and hypertension [36]. Both SLNB and ALND are linked to an increased risk of lymphedema, with around 5% of SLNB recipients and up to 50% of ALND patients experiencing this condition [36]. Furthermore, regional lymph node radiation has been extensively documented as a major risk factor for the development of lymphedema [35, 36].

Cancer-related lymphedema has been associated with anxiety, depression, and low body confidence [35]. Several interventions have been developed to address postmastectomy lymphedema. Complete Decongestive Therapy (CDT) is considered the gold standard treatment for lymphedema, consisting of a two-phase approach [34, 37•]. The initial phase, known as the reduction phase, aims to decrease limb volume and alleviate symptoms. This is accomplished through various interventions, including manual lymphatic drainage (MLD), multiple layer compression bandaging, exercise, and proper skin care [7••, 37•]. Once maximum reduction is achieved, the maintenance phase is initiated. During the maintenance phase, the primary objective is to sustain the reduced limb volume achieved in the reduction phase. This involves transitioning to compression garments, incorporating self-MLD techniques, engaging in regular exercise, and maintaining a diligent skin care regimen [37•]. The maintenance phase plays a crucial role in preserving the outcomes achieved during the reduction phase and preventing the recurrence or exacerbation of lymphedema symptoms [37•]. The use of pneumatic compression devices that apply intermittent pressure to the limbs in combination with CDT may further enhance the effectiveness of MLD [7••]. In more advanced stages, surgical procedures including debulking, lymphovenous anastomosis, and vascular lymph node transplantation have shown promise in reducing the severity of lymphedema [7••, 38] (Table 1).

### Axillary Web Syndrome (AWS)

AWS, also known as cording, is characterized by the presence of a singular taut, narrow cord or multiple cords, approximately 1 mm wide, within the subcutaneous tissue of the axilla. These cords extend downwards, along the medial or medial-volar surface of the upper arm, and in certain instances, can also be observed along the lateral chest wall [3, 5, 39] (Fig. 1). The palpable cord tightens and causes pain, particularly during shoulder abduction, significantly limiting shoulder ROM [3, 5, 39]. AWS typically occurs 2–8 weeks after breast cancer surgery and resolves spontaneously within 3 months, although some cases can persist for years. Recent studies indicate that AWS can develop as well as recur within months to years after surgery [5, 8, 39]. The reported incidence of AWS varies widely, from 6 to 86%, partly due to misdiagnosis and confusion with scar tissue [8, 39, 40].

Although the pathogenesis is unclear, cording is believed to be caused by lymphatic vessel and tissue damage during procedures like SLNB and ALND, commonly performed alongside mastectomy [5, 40]. ALND surgeries have a higher incidence of cording (36%-72%) compared to SLNB surgeries (11%-58%), and patients with a prior or concurrent mastectomy are at the highest risk of developing AWS [39]. Other factors associated with a higher incidence include lower BMI, younger age, higher education, frequent exercise, increased number of lymph nodes removed, extensive surgery, and adjunctive chemotherapy or radiation therapy [39, 41]. AWS may also be associated with an increased risk of postmastectomy lymphedema, with patients experiencing AWS having a 44% higher likelihood of developing this breast cancer-related lymphedema [8].

Physical therapy plays a crucial role in AWS treatment, focusing on exercises to improve flexibility, strength, ROM, and abduction of the affected limb [41]. Licensed practitioners provide in-clinic treatments including myofascial release, soft tissue mobilization, cord manipulation, and stretching while the arm is abducted, specifically focusing on softening the cord [40, 41]. During soft tissue mobilization, it is not uncommon for cords to spontaneously break [5]. Analgesics, NSAIDs, and proangiogenic drugs are used to manage pain, and when combined with physical exercise, analgesics may expedite recovery [41]. Surgical intervention is reserved for severe cases to remove fibrous cords, but it is generally not recommended due to the increased risk of edema [41] (Table 1).

## Breast Reconstruction Considerations

The literature presents mixed evidence regarding the influence of breast reconstruction on functional impairments. While some studies indicate a heightened risk of impairments when reconstruction is performed alongside mastectomy, others report no significant increase in risk. Limited data are available that directly compare the rates of these impairments based on the type or timing of reconstruction [37•].

A recent systematic review by Guliyeva et al. suggests that implant-based breast reconstruction does not increase the risk of PMPS when compared to other surgical techniques or mastectomy alone [42]. Among the eleven publications included in the review, most reported no elevated risk of PMPS following implant-based reconstruction, and some studies even suggested a potential lower risk of chronic pain with this approach [42]. However, other studies suggest that tissue expander/implant-based reconstruction may increase the likelihood of PMPS [43]. Additionally, data indicate that both implant-based and autologous breast reconstruction techniques may have the ability reduce the risk of breast cancer-related lymphedema [37•].

Concerns have been raised regarding "breast implant illness," which refers to a constellation of symptoms patients attribute to their breast implants including fatigue, chest pain, hair loss, headaches, chills, photosensitivity, skin rashes, and persistent pain [44•]. Despite its popularity on social media, there is a lack of evidence supporting these claims. Extensive data, backed by the FDA, reaffirms the safety of silicone breast implants. Currently, no conclusive evidence exists to support the existence of "breast implant illness" [44•].

Abdominally-based autologous reconstruction is a commonly utilized technique for breast reconstruction, involving the use of abdominal tissue [3, 45]. In the transverse rectus abdominis myocutaneous (TRAM) flap procedure, the breast is reconstructed using a portion of the rectus abdominis muscle, along with skin and fat from the lower abdomen. In contrast, the deep inferior epigastric perforator (DIEP) flap procedure preserves the abdominal muscles and utilizes only the skin and fat from the lower abdomen [45]. A prospective study by Roth et al. revealed increased pain after two years in patients who underwent TRAM/DIEP surgeries compared to those who had tissue expander/implant-based reconstruction [46]. According to a retrospective analysis conducted by Yang et al., it was observed that latissimus dorsi (LD) flap reconstruction resulted in a reduction in shoulder muscle strength, while implant-based and abdominally-based reconstruction did not have any significant impact on shoulder muscle strength [47]. Furthermore, studies have shown that LD reconstruction is associated with the highest occurrence of overall shoulder morbidity, followed by tissue expander/implant-based reconstruction, with the lowest rate observed among DIEP patients [48].

Further research is essential to comprehensively evaluate the potential risks associated with breast reconstruction, aiming to address patient concerns, alleviate anxiety, and facilitate informed decision-making. It is crucial for future studies to distinguish between different types and timing of reconstruction to provide more precise and tailored insights.

## Conclusion

This article presents a comprehensive overview of the prevalent functional impairments encountered by breast cancer survivors undergoing mastectomies, along with the interventions designed to effectively mitigate these challenges. The key findings emphasize the widespread occurrence of postmastectomy functional impairments, encompassing neuromuscular, musculoskeletal, and lymphovascular complications. A thorough understanding of these categories is imperative for the development of tailored interventions and optimized treatment plans for patients, thereby improving QoL. Central to the management of functional impairments among postmastectomy individuals is the pivotal role played by cancer rehabilitation, coupled with other strategic interventions. This holistic approach encompasses a diverse array of therapeutic modalities, exercises, and support services. Its objective is to effectively address the physical, psychological, and functional challenges experienced by breast cancer survivors, thereby promoting their recovery, rehabilitation, and overall well-being.
