# Correction to: Postmastectomy Functional Impairments

**DOI:** 10.1007/s11912-023-01481-7

**Published:** 2023-12-11

**Authors:** Eden Marco, Gabrielle Trépanier, Eugene Chang, Emma Mauti, Jennifer M. Jones, Toni Zhong

**Affiliations:** 1 Temerty Faculty of Medicine, University of Toronto, Toronto, ON Canada; 2 Department of Medicine, Division of Physical Medicine & Rehabilitation, University of Toronto, Toronto, ON Canada; 3 Department of Supportive Care, Cancer Rehab & Survivorship Program, Princess Margaret Cancer Centre, Toronto, ON Canada; 4 Multisystem & Musculoskeletal Rehabilitation Program, Toronto Rehabilitation Institute, Toronto, ON Canada; 5 Cancer Rehabilitation and Survivorship Program, Department of Supportive Care, Princess Margaret Cancer Centre, Toronto, ON Canada; 6 Department of Psychiatry, University of Toronto, Toronto, ON Canada; 7 Division of Plastic and Reconstructive Surgery, Department of Surgery, University of Toronto, Toronto, ON Canada

**Correction to: Current Oncology Reports**

10.1007/s11912-023-01474-6

The authors would like to add the following ‘Acknowledgements’ statement.

**Acknowledgements**

Figure 1 illustrated by Diana Kryski.

